# Pulmonary Embolism and Intracardiac Type A Thrombus with an Unexpected Outcome

**DOI:** 10.1155/2017/9092576

**Published:** 2017-04-02

**Authors:** João Português, Lucy Calvo, Margarida Oliveira, Vítor Hugo Pereira, Joana Guardado, Mário Rui Lourenço, Olga Azevedo, Francisco Ferreira, Filipa Canário-Almeida, António Lourenço

**Affiliations:** ^1^Cardiology Department, Hospital Senhora da Oliveira, Guimarães, Portugal; ^2^Escola de Ciências da Saúde, Universidade do Minho, Braga, Portugal; ^3^Cardiology Department, Centro Hospitalar de Leiria, Leiria, Portugal

## Abstract

Detection of right heart thrombi (RHT) in the context of pulmonary thromboembolism (PE) is uncommon (4–18%) and increases the risk of mortality beyond the presence of PE alone. Type A thrombi are serpiginous and highly mobile and are thought to be originated from large veins and captured in-transit within the right heart. Optimal management of RHT is still uncertain. A 79-year-old woman, with a history of recent total hysterectomy with adnexectomy and a Wells procedure, presented to the emergency department following an episode of syncope. Computed tomography revealed bilateral PE and the presence of a right atrial thrombus. Transthoracic echocardiography demonstrated a free-floating type A thrombus in the right atrium, protruding into the right ventricle, and signs of pulmonary hypertension and right ventricle dysfunction. Considering the recent surgery and clinical stability, treatment with heparin alone was decided. Subsequent clinical improvement was observed and echocardiographic follow-up revealed complete thrombus dissolution and complete recovery of right ventricle function. Most authors recommend treatment of PE with RHT with thrombolysis or embolectomy followed by anticoagulation, although evidence is scarce. Individual risk of hemorrhage and operatory-related mortality should be taken into account when defining the treatment strategy especially when benefit is not firmly established.

## 1. Background

Venous thromboembolism, including pulmonary embolism, is a common disease that carries significant morbidity and mortality. The presence of heart right thrombi (RHT) in the absence of atrial fibrillation, structural heart disease, or catheters in situ is rare and almost exclusively found in the presence of clinical manifestations of pulmonary embolism (PE). In view of the reported high mortality, it constitutes a medical emergency and requires immediate treatment. Although there are different treatment options for PE, the optimal management of right ventricular thrombi is still uncertain.

We report a case of a patient with a free-floating type A thrombus with a favorable outcome with a conservative approach and review the related literature [1].

## 2. Case Report

A 79-year-old woman presented to the emergency department following an episode of syncope. She had undergone a total hysterectomy with adnexectomy and a Wells procedure for complete rectal prolapse 2 weeks before and had history of resting dyspnea in the previous week.

Upon examination she was hypotensive (85/49 mmHg), tachycardiac (110 bpm), and tachypneic (25 bpm). Oxygen saturation was 98% on room air. The electrocardiogram demonstrated sinus tachycardia with no other changes. Fluid therapy was promptly initiated and the patient rapidly became normotensive.

**Figure 1:**
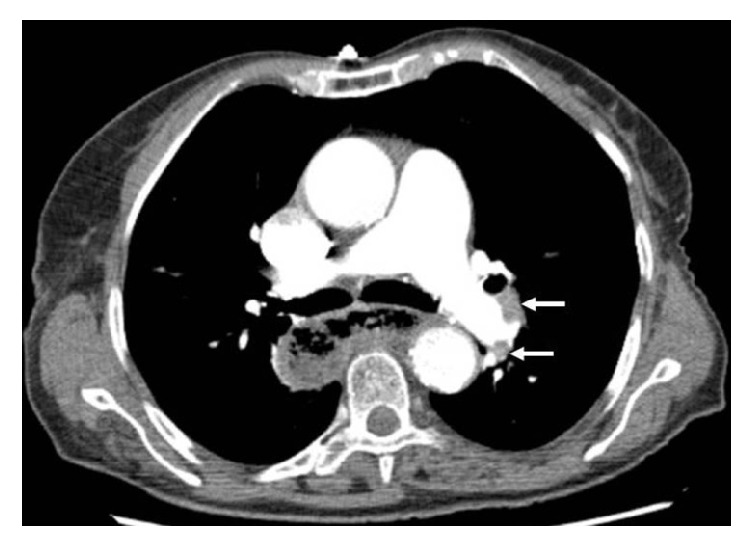
Computed tomography angiography showing proximal pulmonary emboli (white arrows).

**Figure 2:**
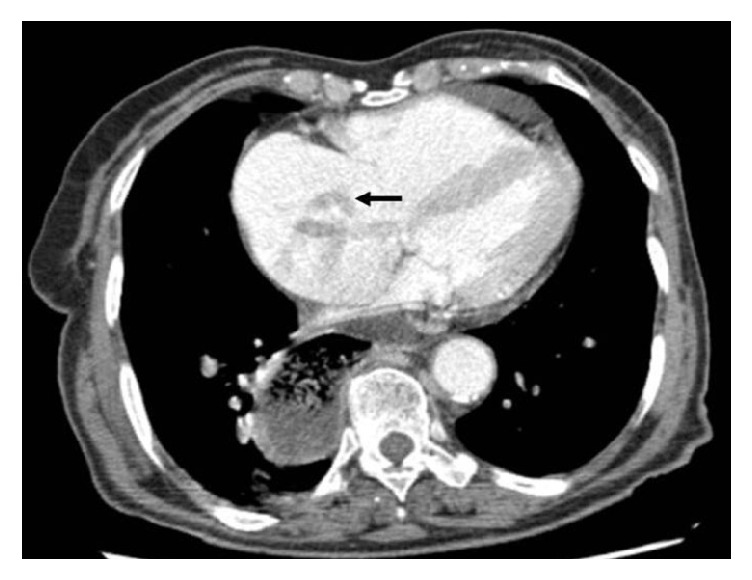
Computed tomography angiography revealing right heart chamber dilatation and the presence of a curled worm-like thrombus (type A) in the right atrium.

Urgent computed tomography angiography revealed bilateral PE (Figure 1) and dilatation of the right ventricle and suggested the presence of a long thrombus on the right atrium (Figure 2).

**Figure 3:**
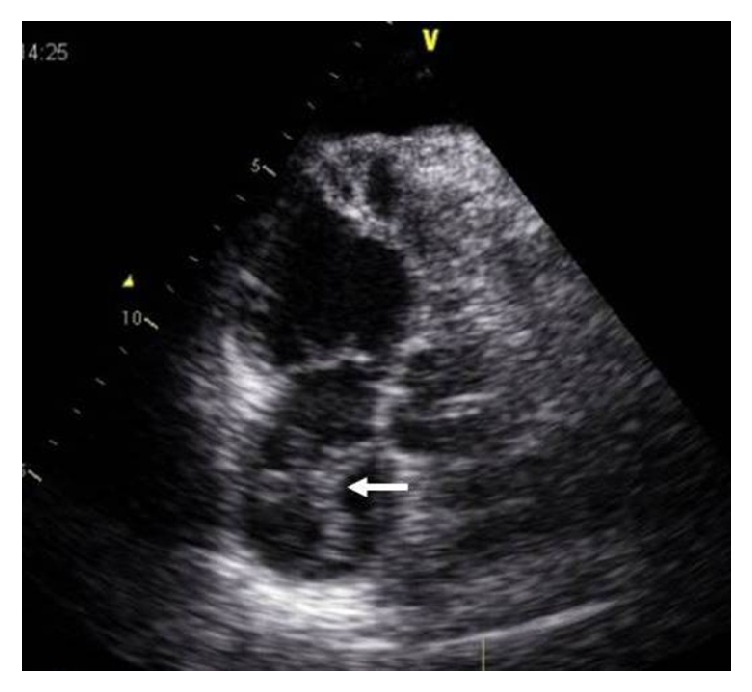
Transthoracic echocardiography apical view showing a mobile thrombus in the right atrium in systole (white arrow).

**Figure 4:**
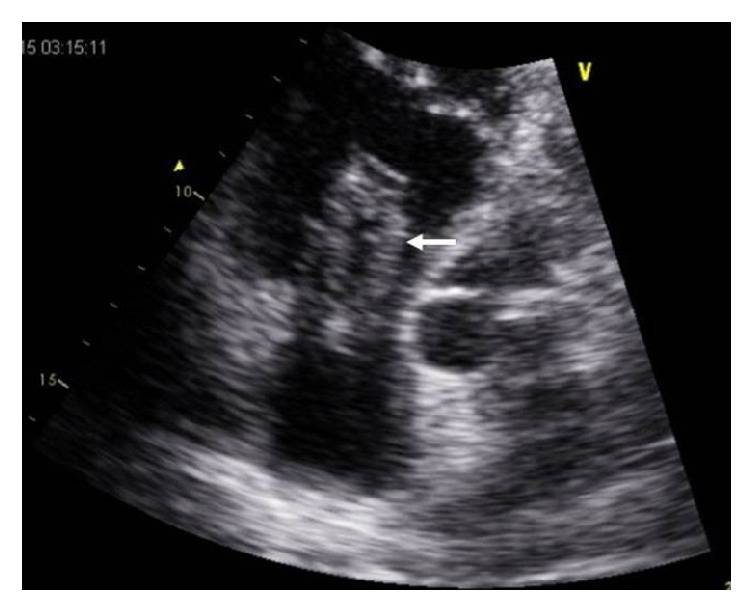
Transthoracic echocardiography apical view showing a mobile thrombus protruding to the right ventricle in diastole (white arrow).

Transthoracic echocardiography documented a big worm-like mass floating in the right atrium, protruding through the tricuspid valve into the right ventricle in diastole (see video 1 in Supplementary Material available online at https://doi.org/10.1155/2017/9092576). Severe dilatation of the right heart chambers (right ventricle: 41 mm), significant tricuspid regurgitation, and moderate pulmonary hypertension (estimated pulmonary artery systolic pressure: 58 mmHg) were also documented (Figures 3 and 4).

Considering the high risk of hemorrhage with the recent surgery and the patient's clinical stability, conservative treatment was decided with no thrombolytic therapy or surgical embolectomy. Anticoagulation with intravenous unfractionated heparin was started immediately with a 5000 IU bolus, followed by an infusion of 18 IU/Kg targeting a therapeutic aPTT level between 46 and 70 seconds. At admission in the cardiac intensive care unit she presented mild dyspnea at rest with no other symptom and her blood pressure was stable (systolic BP > 100 mmHg).

The following day the patient's symptoms had improved, with no resting dyspnea, although the echocardiography assessment showed no improvements. On the third day the patient became completely asymptomatic. The echocardiographic evaluation on the fourth day of hospitalization showed no signs of intracardiac thrombus, and partial recovery of right ventricle function with free-wall hypokinesis and mild tricuspid regurgitation with no signs of pulmonary hypertension was observed.

**Figure 5:**
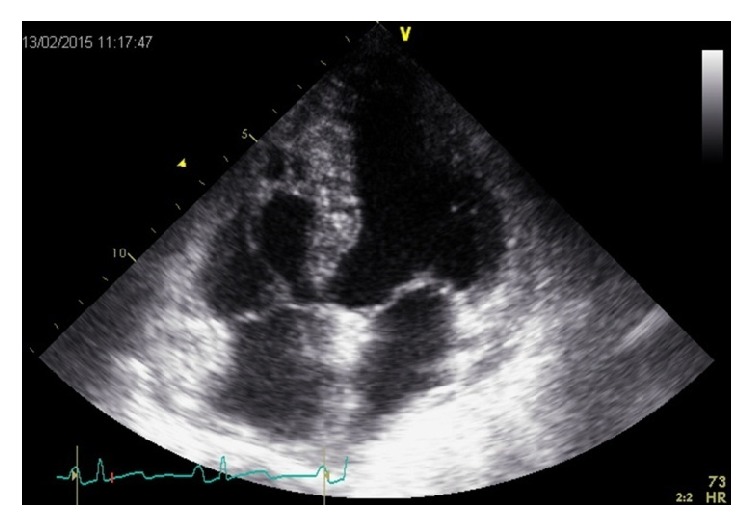
Transthoracic echocardiography apical four-chamber view at discharge with no evidence of intracardiac thrombus or right ventricle dysfunction.

At discharge (9 days after admission) the transthoracic echocardiography was unremarkable, with normal right ventricle function (Figure 5). The patient was discharged from the hospital on anticoagulation with warfarin.

## 3. Discussion

In the setting of PE, echocardiography is currently indicated for the diagnostic work-up in suspected high risk PE and for prognosis assessment in intermediate-risk patients [2]. Echocardiography is considered the examination of choice for the detection and morphology assessment of RHT, although computed tomography has also been shown to be an accurate diagnostic tool in this setting [3]. Bedside echocardiography is an invaluable tool in the management of these patients, allowing serial assessment of right ventricular chambers size and function and of changes in thrombus size or morphology.

Despite the increase in the diagnosis with the widespread use of echocardiography, free-floating RHT are still considered a rare finding. They can be identified in less than 4% of unselected patients with PE, but their prevalence may reach 22% in high risk patients [3–6].

Three patterns of RHT have been described. Type A thrombi are morphologically serpiginous, highly mobile, and associated with deep vein thrombosis and PE. It is hypothesized that these clots embolize from large veins and are captured in-transit within the right heart. Type B thrombi are nonmobile and are believed to form in situ in association with underlying cardiac abnormalities while type C thrombi elicit intermediate characteristics of both type A and type B [7]. Our patient presented a serpiginous thrombus moving through the tricuspid valve to the right ventricle compatible with a type A thrombus.

Mobile RHT are associated with RV dysfunction and higher early mortality beyond the presence of PE alone. The presence of RHT at the time of acute PE was found to predict all-cause death, PE-related death, and recurrent venous thromboembolism, particularly in patients without haemodynamic compromise [6].

A meta-analysis including all reported cases in the literature up to 2002 reported a 27% mortality rate, with 100% mortality in untreated patients [8]. Observational studies also report high early mortality rates, ranging from 29% to 50% [4, 9, 10]. More recent meta-analysis data suggest a slightly lower mortality rate of 16.7% [11].

However, it is still unclear whether RHT are a direct cause or just an indicator of adverse outcomes. In the Right Heart Thrombi European Registry, short-term prognosis was associated with clinical and haemodynamic consequences of PE and not RHT characteristics such as size, morphology, or mobility [12].

In view of the reported high mortality, the coexistence of PE with RHT is regarded as a medical emergency and requires immediate treatment. Contemporary treatment modalities for PE vary, ranging between heparin alone, thrombolysis, catheter-directed therapy, and surgical embolectomy. However, the optimal management of PE associated RHT remains unclear owing to the low number of cases and the lack of randomized controlled trials.

The haemodynamic benefits of thrombolysis in patients with PE are well established and it is currently recommended for high risk and selected intermediate-high risk patients [2]. Thrombolytic treatment has the potential to dissolve the clots at three locations: the intracardiac thrombus, the pulmonary embolus, and the venous thrombosis. The benefit of using systemic thrombolysis can also be attributed to its easy availability, rapid bedside initiation, and simplicity of treatment. Good outcomes with thrombolysis were reported in some small series of patients with RHT [13, 14]. Torbicki et al. proposed that the favorable result after thrombolysis could be related to the shorter delay between the presumed onset of symptoms and hospitalization in patients where pulmonary embolism was associated with mobile clots in the right heart (2.2 versus 4.5 days) [4]. In one case series, half of the clots disappeared within 2 hours of thrombolysis, whereas the remainder disappeared within 12 to 24 hours. This delayed disappearance of the thrombi supports the decision to defer surgery after thrombolysis until at least 24 hours [13].

Surgical embolectomy with exploration of the right heart chambers and pulmonary arteries under cardiopulmonary bypass is another treatment option [2]. However, it is not readily available in many centers and it carries the risk of inherent delay of a few hours, general anesthesia, cardiopulmonary bypass, and the inability to remove coexisting pulmonary emboli beyond the central pulmonary arteries. It should be considered particularly for cases in which thrombolysis is contraindicated or if thrombolysis is ineffective or in patients with patent foramen ovale with potential for systemic embolization.

Emerging catheter-directed therapies for PE include percutaneous catheter-directed thrombolysis or high-frequency ultrasound exposure near the surface of the clot; endovascular mechanical thrombectomy using fragmentation and a capture device; and endovascular aspiration of the clot directly from within the atrium, ventricle, or pulmonary arteries [15–17]. These methods are also promising in patients with RHT with some successful cases reported [18–21]. However evidence is still scarce and there is still a lack of general availability and expertise.

Anticoagulation is recommended for virtually all PE patients and should be promptly initiated while awaiting the results of diagnostic tests [2]. In the presence of an intracardiac thrombus it can be used alone as first-line therapy or as an adjunctive following other interventions. The use of isolated parenteral anticoagulation is sometimes dismissed in patients with RHT because it is thought to be potentially hazardous as the thrombi may embolize to the already compromised pulmonary circulation, although thrombolysis may also pose similar risks [8]. However, its use as a first-line therapy in these patients is proposed in stable patients, especially when there is a high bleeding risk [9].

Few studies have analyzed the differential impact on mortality of these therapies. Older meta-analysis data showed no differences among available treatment options [22]. More recently, Rose et al. reviewed 177 cases presenting with RHT described on literature and described an improved survival rate with thrombolytic therapy that was statistically significant when compared to either anticoagulation therapy or surgery [8].

However, case series of consecutive patients comparing these therapeutic approaches failed to find significant differences on mortality, although the results are most likely limited by the small number of patients included [4, 9, 10]. More recent data using propensity scores to compare reperfusion therapy to anticoagulation alone found no significant difference in mortality and bleeding, with a higher risk of recurrence with reperfusion therapy [23].

The presence of a right heart thrombus is rare, and it is unlikely that a randomized trial with two or three different treatment arms would be performed in the near future. Thus, choice of therapy is based on the physician's discretion and clinical judgment and based on availability and patient factors which often preclude the development of one-size-fits-all treatment algorithms.

In this case, a favorable course with complete thrombus dissolution and right ventricle function recovery was observed with a conservative approach with heparin alone. This highlights the importance of taking into account individual risk of hemorrhage and operatory-related mortality and considering less invasive strategies such as isolated anticoagulation in these patients.

## 4. Conclusion

Although it is widely recognized as an ominous finding, the existing literature does not offer a clear consensus for management of PE with coexisting mobile intracardiac thrombus. Existing evidence suggests the superiority of thrombolysis over anticoagulation alone, and most authors advocate immediate treatment with thrombolysis and/or embolectomy followed by effective anticoagulation with heparin, even though there are no prospective randomized trials to support this decision. Percutaneous treatments may play an important role for the management of patients with RHT in the future, but evidence is still lacking.

This case illustrates the difficulty in the management of such high risk patients in the absence of hard evidence and highlights the importance of taking into account individual risk of hemorrhage and operatory-related mortality when defining the treatment strategy. Avoiding high risk procedures should always be carefully considered when benefit is not firmly established* (primum non nocere)*.

## Supplementary Material

Transthoracic echocardiography subcostal view showing a free-floating worm-like mass in the right atrium protruding through the tricuspid valve into the right ventricle in diastole. Signs of pulmonary hypertension such as right heart chamber dilatation and interventricular septum displacement toward the left ventricle are also observed.

## Abbreviations

RHT: Right heart thrombi
PE: Pulmonary embolism.
